# *φ*-evo: A program to evolve phenotypic models of biological networks

**DOI:** 10.1371/journal.pcbi.1006244

**Published:** 2018-06-11

**Authors:** Adrien Henry, Mathieu Hemery, Paul François

**Affiliations:** Physics Department, McGill University, Montreal, Québec, Canada; Hebrew University of Jerusalem, ISRAEL

## Abstract

Molecular networks are at the core of most cellular decisions, but are often difficult to comprehend. Reverse engineering of network architecture from their functions has proved fruitful to classify and predict the structure and function of molecular networks, suggesting new experimental tests and biological predictions. We present *φ*-evo, an open-source program to evolve *in silico* phenotypic networks performing a given biological function. We include implementations for evolution of biochemical adaptation, adaptive sorting for immune recognition, metazoan development (somitogenesis, hox patterning), as well as Pareto evolution. We detail the program architecture based on C, Python 3, and a Jupyter interface for project configuration and network analysis. We illustrate the predictive power of *φ*-evo by first recovering the asymmetrical structure of the *lac* operon regulation from an objective function with symmetrical constraints. Second, we use the problem of hox-like embryonic patterning to show how a single effective fitness can emerge from multi-objective (Pareto) evolution. *φ*-evo provides an efficient approach and user-friendly interface for the phenotypic prediction of networks and the numerical study of evolution itself.

This is a *PLOS Computational Biology* Software paper.

## Introduction

Increasing availability of massive datasets combined to improvement of computational power have triggered the current boom of machine learning [1]. While methods such as deep learning are extremely powerful for predictions, they are nevertheless hard to comprehend, and thus are ill-adapted for the fundamental study of the processes generating those data. This led to the development of more parsimonious inverse problem approaches: for instance the “automatic statistician” relies on the combination of predefined kernels [2] to generate explicit models of data. Despite their potential for the understanding of biological mechanisms, few similar methods are currently available or tailored for quantitative biology. Bayesian criteria have been used to generate non-linear models with few parameters [3] but applications to biological problems are still in their early days. Enumeration of all possible networks compatible with a phenotype [4] are naturally limited in scope by the combinatorial explosions of interactions. This is not a problem for evolutionary algorithms that can generate solutions of arbitrary complexity with an increase in genome size [5, 6]. Such approaches further allow to focus on biologically relevant questions such as development [7–10], allostery [11, 12] or more general issues such as the impact of biochemical constraints on biological functions [13].

**Fig 1:**
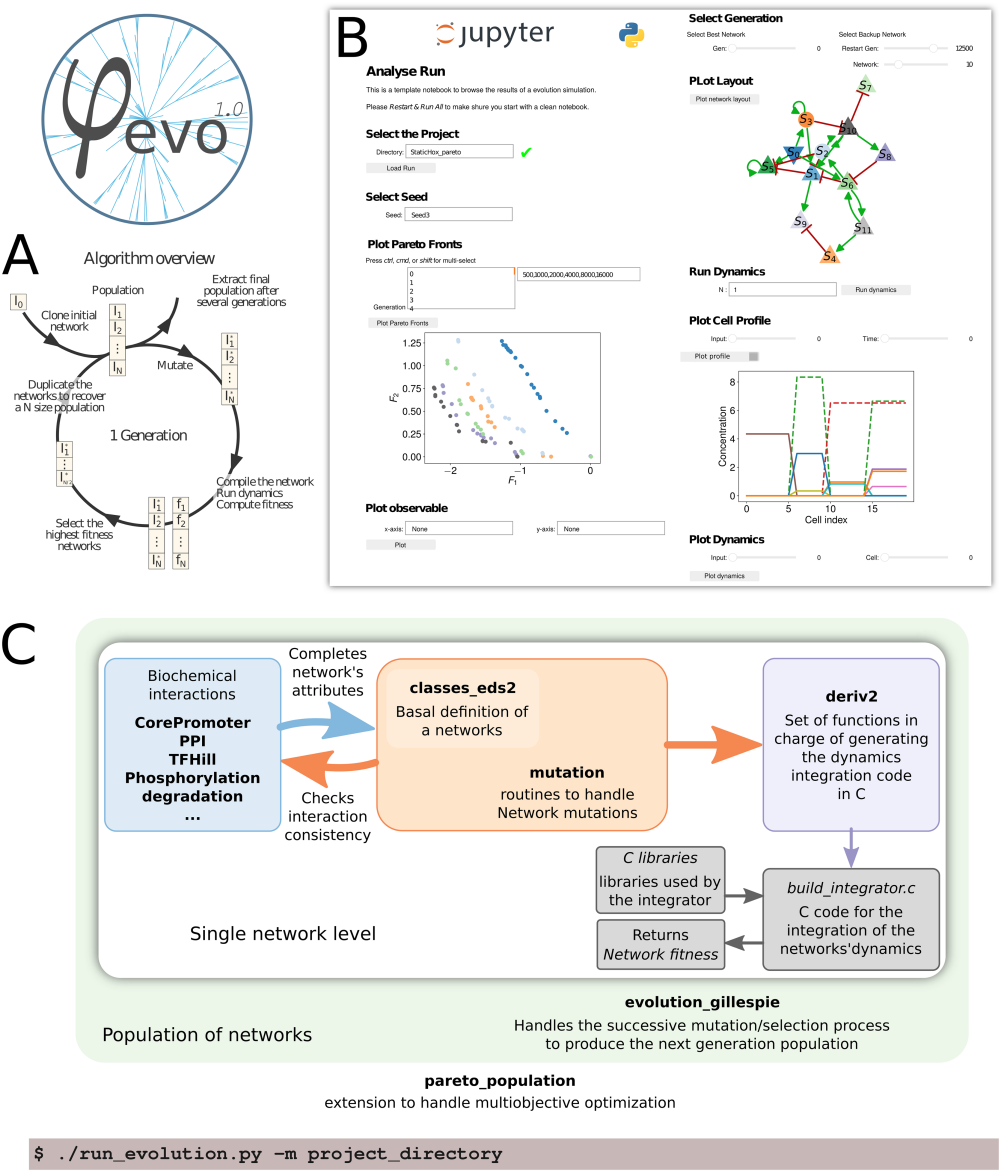
Program structure. (A) A schematic representation for the core loop of a typical evolutionary algorithm. By analogy with Darwinian evolution, each loop is called a generation and implements reproduction, selection and mutation. (B) We provide a Jupyter notebook template for the analysis of the *φ*-evo results. (C) A schematic representation of the program structure. The algorithm is called through the run_evolution.py script that reads an initialization.py file to set up the various parameters for the current project (fitness and input files, mutation rates, etc…). It relies on the evolution_gillespie module that implements the population class and the various method used for the evolutionary procedure. Each individual in the population being a mutable_network as described in the mutation and classes_eds2 modules. The various possible interactions are implemented in separate modules (CorePromoter, PPI, etc.) while the integration of the set of differential equations corresponding to each network relies on the deriv2 module and the associated C code files.

In this report, we describe *φ*-evo, an evolutionary program that evolves phenotypic models of cellular processes. Given a fitness function encoding coarse-grained phenotypes, *φ*-evo randomly combines and optimizes biologically inspired kernels using a genetic algorithm, Fig 1A. *φ*-evo can be applied to problems of different nature thanks to flexible kernel definitions, modeling processes like transcription, ligand receptor/ protein-protein interactions, kinetic proofreading or phosphorylations, allowing to generate (validated) predictions in individual cells or in multicellular contexts. *φ*-evo is open-source, relies on commonly used tools (Python 3, gcc) and results can be visualized via a Jupyter interface Fig 1B.

The code repository includes all tools allowing for evolution of single cell and multicellular/developmental problems, and several examples are included (see details below). Jupyter notebook templates are also included to configure a new project and visualize results. Here we further illustrate the power of *φ*-evo on two simple problems: first we reproduce the *lac* operon asymmetrical architecture using a simple symmetrical objective function, second we show on a developmental example how a well-defined effective fitness function emerges from multi objective Pareto evolution.

## Design and implementation

### Algorithm overview

*φ*-evo relies on an evolutionary algorithm acting on a population of networks, to explore the space of possible biochemical networks implementing a particular function of interest [14].

There are two levels in the algorithm: the *individual level* (white box on Fig 1C) where genotype, phenotype and evolutionary fitness of individuals are defined and computed, and the *population level* (green boxes on Fig 1C), where the evolutionary process is actually performed. All objects (biochemical interactions, networks, populations) are defined using customized Python classes, and explicit numerical calculations are compiled on the fly and performed in C for faster performance. A complete documentation for all Python classes enriched with examples is included and can be automatically (re)generated.

### Individual level

Individual networks in a population are represented as Python objects called Mutable_Network. This class encodes the topology of the network considered, the different parameters corresponding to biochemical species and their interactions, and all the methods that will be used at the individual level, including fitness computation and mutations of the network.

#### Network representation

Each biochemical network is encoded with a graph where nodes represent either the biochemical species or the interactions between them.

Species nodes usually correspond to proteins. They are described through a list of tags (a complete list of tags is given in Supplement), each one indicating a biochemical property (e.g. only 'Degradable' species are susceptible to be degraded), and associated parameters if needed (e.g. the degradation rate). A second type of node, TModule allows to model DNA transcriptional modules (or enhancers).

The Interaction objects correspond to the various chemical reactions allowed between Species or TModule, and play similar roles to kernels in other machine learning approaches such as [2]. Derived classes are defined for each possible biochemical interaction. We include by default generic interactions such as transcription (CorePromoter), transcriptional regulations (TFHill), protein-protein interactions (PPI) and phosphorylations (Phospho). We also include an extension where problem-specific interactions are defined such as phosphorylation cascades of immune receptors [15].

Species and Interaction form a bipartite graph that is encoded using the networkX package [16]. The Networks/ directory gathers files for the Mutable_Network definition; the most important ones being the basic network structures in classes_eds2.py and the mutation methods in mutation.py.

#### Computing a network phenotype

A Mutable_Network is translated into a set of differential equations. By default, we use classical biochemical kinetics to account for the various interactions, e.g. mass-action laws for protein-protein interactions, or Hill functions for transcriptional interactions [17]. We also assume transcription by multiple activators acts akin to an “OR” gate. Those specific modelling choices can be easily changed by the addition of customized Interaction classes. As mutations add or remove new nodes in the network, the corresponding equations are generated and integrated on the fly. This is performed by the deriv2.py module that takes a single network, generates, compiles and runs a network-specific C program to compute its fitness.

Practically, various C headers are pre-encoded, imposing initial conditions of the simulations, geometry of the problem (for multicellular problems), and numerical integrator used (default tools for single cell problems and embryo modeled as a line of cells are included). The C headers are shared by all networks of a population in a given set of simulation. Within one evolutionary simulation, the only difference between networks is at the level of differential equations encoded, in the specific C function corresponding to time derivatives. Simulations presented here are run in a deterministic mode, and both Euler and a Runge-Kutta integrators are available in the program. An option to run equations in a stochastic mode using *τ*-leaping algorithm (a biochemical numerical generalization of the Langevin equation [18]) is also included.

The fitness function used for selection is problem-specific and needs to be predefined by the user in corresponding C functions. Defining a “good” fitness is not a trivial problem: a too strict fitness could give rise to very rugged evolutionary landscape with many isolated local minima. An efficient fitness should orient evolution towards working solutions even when the networks are still far from the optimum behaviour. Different examples in the package use coarse-grained fitnesses allowing for fast convergence of the evolutionary simulations.

#### Mutating a network

The module Networks/mutation.py encodes the main evolutionary methods to mutate, integrate, compute the fitness and copy the individual. There are 3 categories of mutations:

- modification of parameters of existing interactions or species
- removal of interactions or species
- addition of new interactions or new species

Those three operators are interaction specific and are thus defined and implemented in the corresponding Python classes. Relative mutation rates are fixed by the user. By default, modifications of parameters consist in simple uniform draw within a predefined range of kinetics, but more involved choices are possible. Removal and addition of interactions change the network topology, and are chosen randomly among possible interactions. Adding or removing species is done jointly with associated TModule and CorePromoter (see Networks/CorePromoter.py), encoding default transcriptional dynamics. We also include some tools to duplicate transcriptional interactions.

### Population level

We evolve a population of networks as defined above using a classical evolutionary algorithm. By default, rounds of selection, expansion and mutation are performed as depicted in Fig 1A.

At each generation, we compute the fitness of each network, and impose a strict elitist selection [19] where the worst half of the population is discarded, and the best half is selected and duplicated. Mutation operators act on every individual in the duplicated half population. Mutation rates are predefined by the user, and by default are rescaled at each generation to maintain on average a fixed number of mutations per network per generation (typically one or two). This prevents uncontrolled mutations due to combinatorial explosion, and fits the phenomenological Drake’s rule (a scaling relation of mutation rates with genome size) observed in real evolution [20].

We also include a Pareto version of our algorithm as proposed in [21]. In summary, Pareto evolution aims at optimizing simultaneously several constraints to circumvent the problem of building an ad-hoc fitness combining those. To rank the networks prior to selection, one sequentially computes so-called Pareto-ranks. The networks of rank 0 are those which are not dominated by any other network on all constraints. Rank 1 networks are only dominated by rank 0 networks, and so on. We then perform elitist selection based on ranks of networks. A fitness sharing option is also available to promote diversity in the population [21].

**Fig 2:**
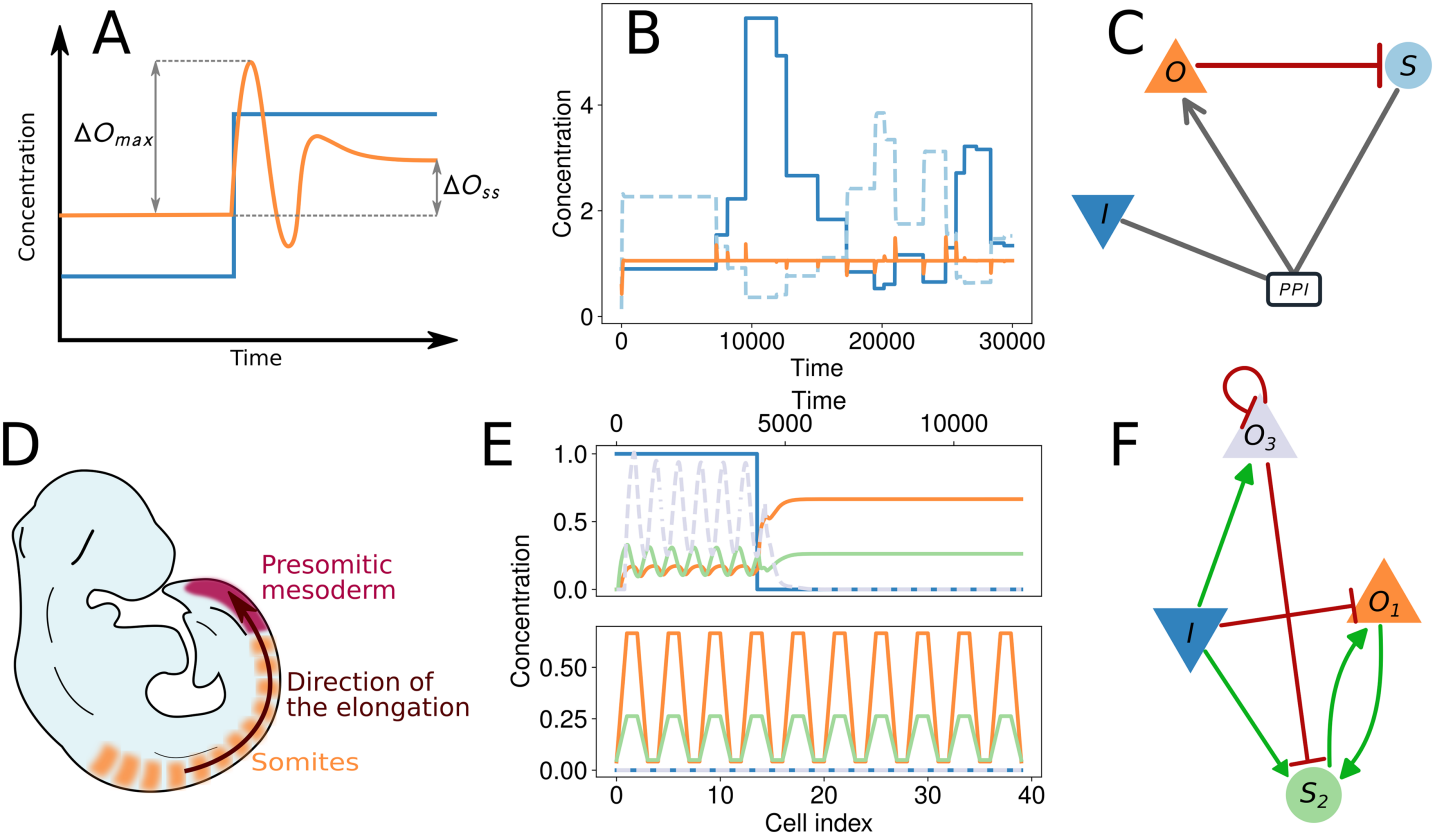
Visualization of the results of *φ*-evo. (A,B,C) Application of *φ*-evo on biochemical adaptation, reproducing results of [22]. Panel (A) describes the fitness, similar to the one in [22]. For ideal adaptation, an output (yellow) should recover its steady state Δ*O*_*ss*_ → 0 after a change of the input (blue). To ensure that the input affects the output, one also wants to maximize Δ*O*_*max*_. (B) presents the behaviour of a network evolved with *φ*-evo, where the output is subject to several changes of the input producing a perturbation of its concentration. The return to steady state is quick. Corresponding network topology is presented in panel (C) and is similar to solutions found in [22] (D,E,F) Application of *φ*-evo to segment formations, reproducing results of [23]. Panel (D) sketches mouse somitogenesis, coupled to embryonic elongation. We use input dynamics modeling elongation and a fitness function counting final number of segments similar to [23]. (E) shows the behaviour of an evolved network, where the system undergoes a bifurcation from an oscillatory to a bistable system when it exits the tail-bud (modelled as the region of high Input) (E-*top*). Depending on its state when leaving, the cell reaches one of two possible steady states. The constant rate of elongation then produces regularly spaced high concentration and low concentration cells (E-*bottom*). See [23] for more details. Corresponding network topology is presented in panel (F).

Since all individual networks can be integrated independently, *φ*-evo can be easily parallelized, and by default multi-threading is used. We routinely run *φ*-evo on modern multi-core machines or small clusters, with population of a few dozens individuals, so that each generation takes at most a few seconds of computation for examples presented here. Running time of the algorithm almost directly scales with the size of the problem studied, and in particular is proportional to the network size and to the number of cells simulated in an embryo. Small networks with few nodes such as the ones displayed on Fig 2 can evolve in less than a couple hundreds of generations and thus a successful run can be obtained in less than half an hour on a modern laptop (not all evolutionary runs are successful, but results presented here are “typical” in the sense that runs are more often succesful than not). More complex simulations have been done for embryonic patterning and typically require several thousands of generations to converge and give bigger networks (e.g. 10 to 15 node-networks were routinely obtained in [9]); this requires running times of several hours to a day.

### Running

Evolutionary simulations in *φ*-evo are run via command line. C files corresponding to a specific evolutionary simulation (such as fitness, initial conditions, etc…) should be provided by the user. Evolutionary parameters (such as mutation rates, initial network) are encoded in a file called initialization.py. Those files should be put together in a common directory (called in the following Simulation) and an evolutionary simulation is started with

# shell

python run_evolution.py -m Simulation

A known difficulty in evolutionary simulations is “code-bloat”, where combinatorial explosion and genetic drift can hinder the core structure of a working network. For this reason, we can also run new evolutionary simulations initialized with an evolved network of interest, while forbidding the addition of new nodes. This isolates the “core” working network, and allows for a better understanding of network dynamics.

### Visualization

We have developed two Jupyter -notebooks to make *φ*-evo more user-friendly. The ProjectCreator notebook allows to define all simulation parameters (e.g. mutation rates) in specific widgets, as well as to define initial topologies for evolution if needed (e.g. to bias evolution with a specific network architecture). The AnalyzeRun Notebook allows to visualize results of simulations in a web browser. This is illustrated in Fig 2 with two simulations reproducing results from [22] and [23]. Users can identify interesting evolved networks, check their topology, simulate them on the fly, and do simple changes such as node removals.

## Results

We include in the *φ*-evo program several complete examples (from simulation parameters to actual results) reproducing published results for a variety of problems in single cells (biochemical adaptation) [22] and developmental contexts (somitogenesis [23], Hox patterning [9]), see Fig 2 for two examples. We also include evolution of “adaptive sorting” in immune recognition [15] as an example of external add-on for the study of problems requiring the definition of new interactions. For a given problem, evolutionary computations typically give several networks with different topologies, see e.g. [22] for a detailed study in the case of biochemical adaptation. In the following, we describe two new evolutionary simulations of biological relevance, to illustrate the power of the algorithm to predict networks and the emergence of non trivial evolutionary dynamics.

### *lac* operon

**Fig 3:**
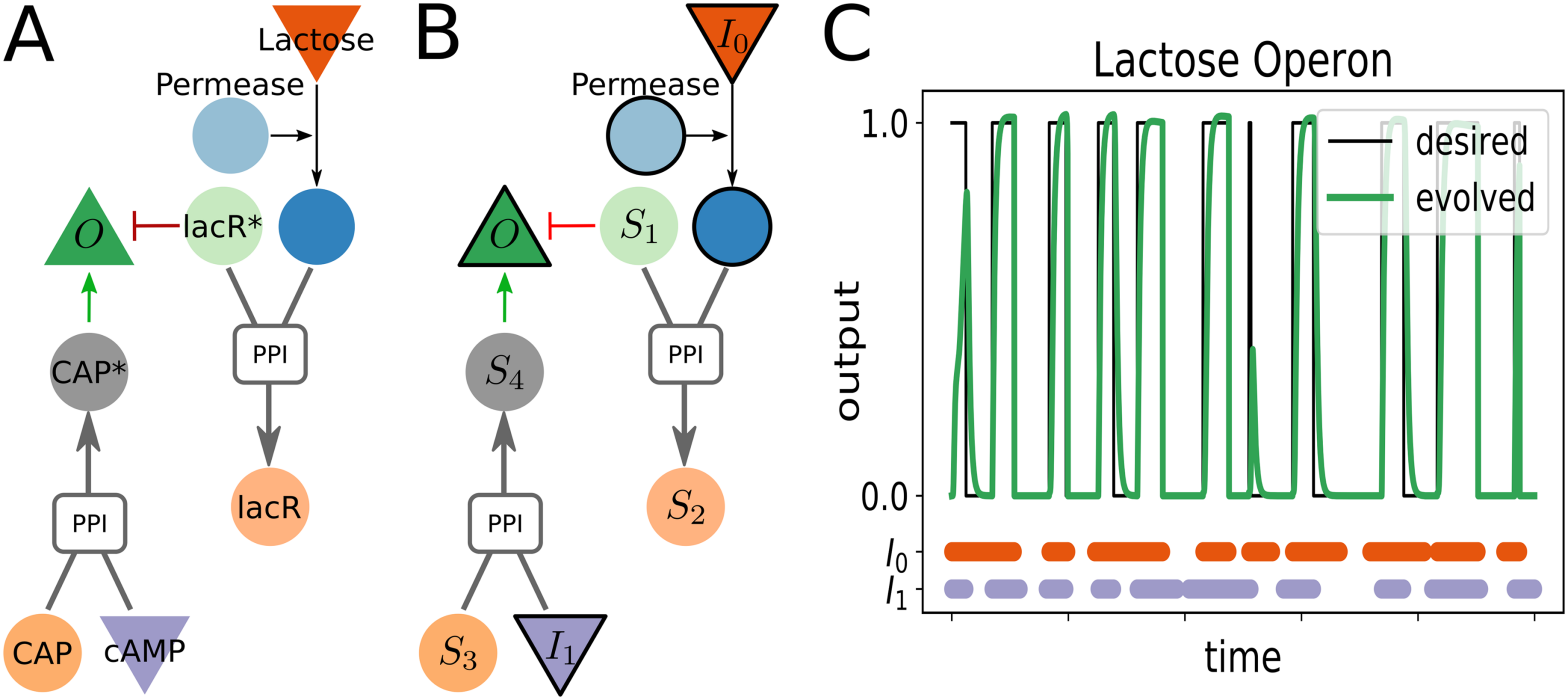
**A** Scheme of the known regulatory network for the *lac* operon. The lac Repressor is inactivated by the presence of lactose which derepress the operon. The cAMP metabolite binds to the CAP protein to activate the operon. **B** Final network found by *φ*-evo after pruning of unnecessary reactions. The similarities with the network **A** are evident. The species with a bold contour are imposed by the definition of our initial network, all other nodes have evolved. **C** Time course of the network presented in **B**, the presence of both Inputs is indicated with the colored bars in the bottom. On the main figure, the output concentration is plotted against time while the desired output is indicated with a black line.

We first use *φ*-evo to recover the regulatory logic of the classical *lac* operon, discovered by Jacob and Monod [24]. The *lac* operon allows for regulation of the enzymes digesting lactose in *E. Coli*. It is activated when two conditions are realized: presence of lactose and absence of glucose. The *lac* Repressor (lacR) represses transcription of the operon while the CAP protein is responsible for its activation. When present, lactose is translocated by permease inside the cell and binds to lacR, which relieves the repression. In the absence of glucose, cAMP is produced in burst, then binds to CAP turning it into an activator. This regulatory mechanism is presented in Fig 3A.

The *lac* operon can be envisioned as a logical binary gate performing the (symmetric) boolean operation “cAMP AND lactose” between those two metabolites. To evolve this function, we initialize a network with an input corresponding to the lactose outside of the cell (denoted *I*_0_), a permease-like enzyme translocating it into the cell, the cAMP species (*I*_1_) and one output coarse-graining all transcriptional targets (*O*). (Bold contours for nodes in Fig 3B). Both inputs are boolean variables that can be be present (*I*_*j*_ = 1 in rescaled units) or absent (*I*_*j*_ = 0), and follow a random dynamics. Importantly we fix the function of the Inputs to be metabolites that cannot activate of repress transcription by themselves, and thus need to act through allosteric binding with other species.

To define the fitness function, we call *T*_on_ the total time when the output should be ‘on’ (i.e. when both inputs are present) and *T*_off_ the remainder where it should be off (i.e. at most one input is present). If we suppose that the output gives a benefit *β* during *T*_on_ and a cost *γ* otherwise, we compute the fitness:

$$\begin{array}{c} \varphi =-\beta \int_{T_{\text{on}}}dt\min\left(O\left(t\right),1.0\right)+\gamma \int_{T_{\text{off}}}dt\min\left(O\left(t\right),1.0\right), \end{array}$$ (1)

and assume this function should be minimized. The min operator ensures that the output simply has to be higher than an arbitrary concentration rescaled to 1 to perform the computation. We also rescale *φ* by setting *β* = 1.

For convergence of the evolutionary process *γ* can not be arbitrary (see details in Supplement). Intuitively, if *γ* is big, it is very costly to get an intermediate step where one input alone controls the Output, which would impinge evolution. Conversely, if *γ* is small, there is no strong incentive to evolve a correct operon because turning the Output on all the time is not costly. We chose $\gamma =\frac{3}{4}$, and checked that the precise value does not change significantly the networks obtained when we vary it.

A representative result of a network found by *φ*-evo is shown in Fig 3B and the behaviour of the network is shown in panel **C**. We display the core network obtained after pruning unnecessary interactions as described in the Implementation section (See Supplements for more details). We can see by direct comparison that the final network of the algorithm appears very close to the structure of the real lac operon displayed in Fig 3A.

While this example might appear simplistic, it shows how even simple simulations can provide insights into the biochemical constraints on evolution. While the objective function puts the two Inputs on equal foot, the evolved network is not symmetrical, presenting regulations via one activation and one repression. This asymmetry actually originates from the default transcriptional grammar implemented: since multiple activators act via an effective “OR” gate, our algorithm can not directly build a transcriptional“AND” gate. The simplest solution is to have activation on one side and titration of the repressor on the other one, which is found by *φ*-evo.

### Emergence of fitness in Pareto optimization

**Fig 4:**
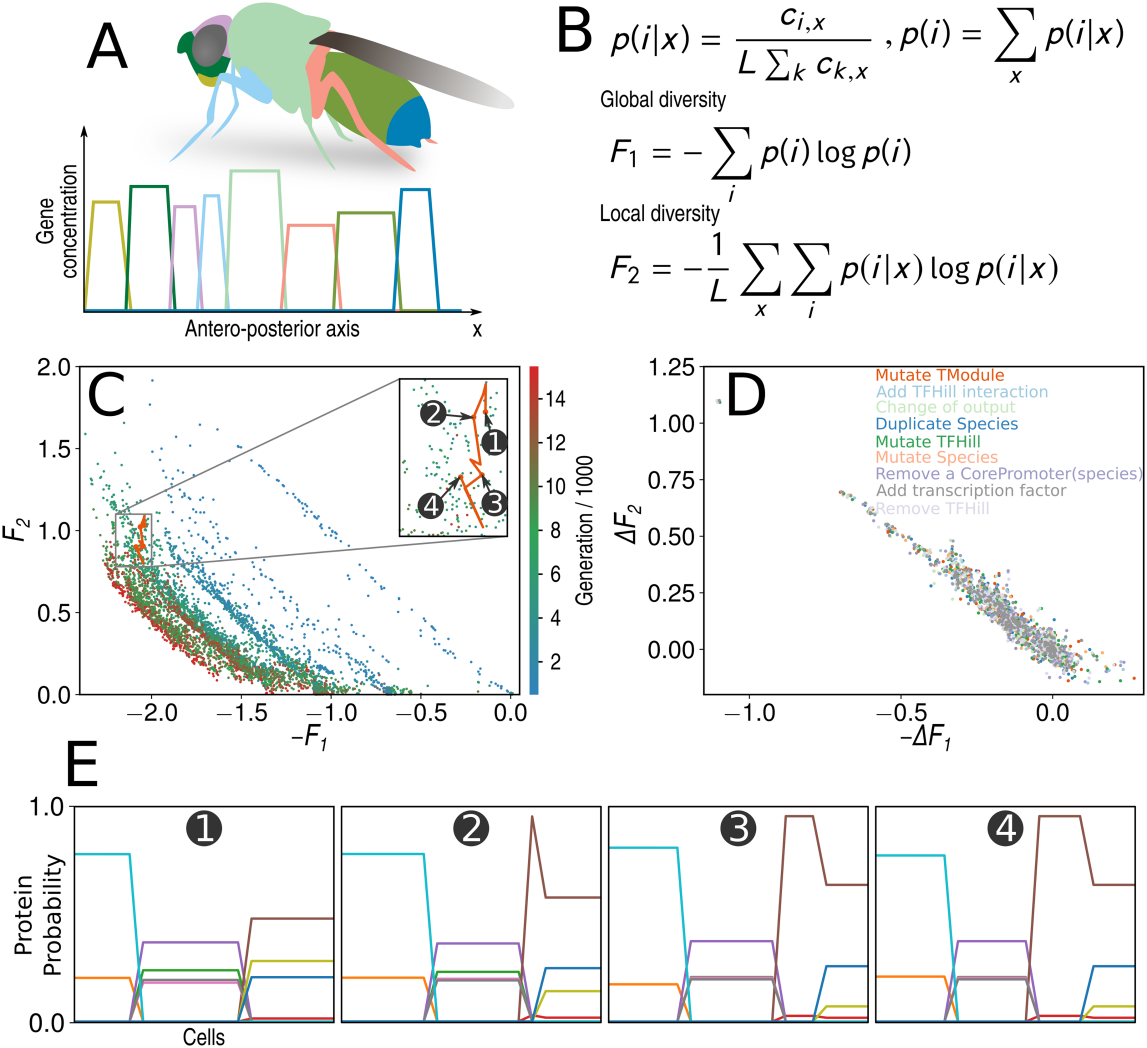
Pareto evolution of embryonic patterning (A) Schematic representation of the problem: we model embryonic patterning as an antero posterior sequence of genes at different positions (B) Fitness definition, where *p*(*i*; *x*) represents the relative fraction of gene *i* expressed at position *x*. *F*_1_ increases with the number of genes expressed within the embryo, while *F*_2_ increases with the number of genes expressed locally at *x*. See [9] for more details on the two fitnesses (C) Pareto fronts for a single evolutionary simulation at different generations (warmer colours correspond to later generations) aiming at maximizing *F*_1_ and minimizing *F*_2_ simultaneously. Inset corresponds to the evolutionary trajectory studied in panel E (D) Changes of functions *F*_1_ and *F*_2_ for random mutations within a network population (E) An evolutionary trajectory leading to the definition of a new Pareto front, from 3 to 4 domains. Only the local probability profiles for the steady states of the networks are displayed.

As a second example, we use *φ*-evo for Pareto evolution of embryonic patterning (Fig 4A). In [9], the computational evolution of hox-like patterning was addressed. A **single** combined fitness aimed at optimizing two terms: a global entropy term, maximizing the number of genes expressed within the embryo (*F*1) and a local entropy term, minimizing the number of genes expressed in a given cell to define local identity (*F*2), 4 B. The fitness *F*2 − *F*1, corresponding to mutual information between cell position and gene expression, was initially minimized, leading to the evolution of networks presenting several characteristic of hox-like patterning, including posterior dominance [9].

Here we revisit this problem using multi-objective/Pareto evolution to minimize **at the same time** −*F*1 and *F*_2_. Typical results of these simulations are presented in Fig 4C, where Pareto fronts for different generations are plotted.

The networks we obtain are qualitatively similar to the ones first evolved in [9] (see Supplement). Remarkably, it appears that the Pareto fronts observed during the course of evolution are slopes of constant *F*2 − *F*1, corresponding to the exact mutual information fitness used in [9]. During evolution the Pareto front clearly evolves towards increasing values of mutual information (corresponding to an increase of the number hox-like domains in an embryo [9]).

The choice of fitness function in [9] was in part motivated by the fact that gene duplications, presumably an important driver for evolution, were neutral moves for the chosen mutual information fitness (see Supplement of [9]). Here the logic appears inverted: during evolution, most mutations are (at best) expected to move networks on the Pareto front. This is illustrated in Fig 4D where the relative changes of fitness are displayed for a population of networks following random mutations. We clearly see that many of the mutated networks localize on the slope Δ*F*_1_−Δ*F*_2_ = 0 (in Supplement we generalize a calculation from [9] to explain this effect). This defines the equivalent of a neutral space, but for Pareto evolution: networks moving on the front are not Pareto-dominated by any other network by definition.

If most mutations are on the Pareto front, this raises the question of how a new Pareto front is reached during evolution. Such a (rare) event is presented in Fig 4E, where we have tracked back the ancestors of a network to build its evolutionary trajectory between two Pareto fronts (inset of Fig 4C). One clearly sees how this network incrementally evolves from 3 to 4 hox-line domains, by addition of an internal domain in the right most one, and refinement/shrinkage of all domains. Interestingly this evolution does not occur in a direction orthogonal to the Pareto front, rather the trajectory zigzags with an overall direction corresponding to a small variation in −*F*_1_ (i.e keeping the global entropy constant) while significantly improving *F*_2_ (the local entropy). This fits the idea that evolution here is driven by similar genes expressed in the same domain, then later specializing to define new domains. However, it is also clear that on the short evolutionary time-scale, evolution is not a straight line in a well-defined direction. We see rather complex stochastic dynamics with the interplay of the two constraints, as can be also gathered from the intermingled structure of Pareto fronts at different generations in Fig 4C.

This example thus illustrates how *φ*-evo can be used to study the dynamics of evolution of complex features, and how an effective fitness function can emerge from the simultaneous optimization of several constraints (the latter presumably being more generic in biology than the former [25]). Indeed the fitness rationalized *a priori* in [9] is discovered here *a posteriori* by *φ*-evo. We could imagine situations where the geometry of Pareto fronts varies during generations, so that the shape of the Pareto fronts and the corresponding effective fitnesses would vary as well during evolution. If the geometry of Pareto fronts is very different in different regions in terms of constraints optimized, we could even imagine local populations evolving in very different ways, potentially leading to numerical speciations.

## Summary

*φ*-evo evolves phenotypic networks performing non trivial biological functions. It can be used to uncover functional features of existing biological networks, as well as to study the dynamics of evolution of gene networks e.g. in the case of Pareto optimization. It represents a useful alternative to black-box modeling approaches for the understanding of biological networks and their evolution.

## Availability and future directions

*φ*-evo is available as a Python package hosted on github at the following link: https://github.com/phievo/phievo

The program documentation is hosted on readthedocs: http://phievo.readthedocs.io/en/latest/

A tutorial video is available here (clickable link) to illustrate how to run *φ*-evo on the example of vertebrate segmentation similar to [23]. We include two other tutorial videos illustrating how to use the ProjectCreator Notebook to initialize and bias an evolutionary simulation here (clickable link), and other features such as node removal here (clickable link).

The algorithm was first developed using Python 2 and the current version runs under Python 3. It can be installed locally using pip. It requires only a C compiler such as gcc as an extra dependency. The algorithm has been developed and optimized in a Unix/Linux environment and has been tested on both Mac OS X and Windows. More details on the implementation and on the examples are given in the Supplement.

Future directions will include automatic model reduction using Fitness Based Asymptotic Parameter reduction ($\bar{\phi }$ [26]), implementation of practical problems such as data fitting, and of networks transitory forms similar to [27].

## Supporting information

S1 Text This text gives more details on the algorithm and on the simulations described in the main paper. (PDF)
