# Supplementary material for: φ-evo: A program to evolve phenotypic models of biological networks
Source: PLoS Comput Biol. 2018 Jun 11;14(6):e1006244. doi: 10.1371/journal.pcbi.1006244 (PMC6013240; doi:10.1371/journal.pcbi.1006244)
Supplement: S1 Text — (PDF) [file pcbi.1006244.s001.pdf]

S1 Text

June 5, 2018

1 Tests and examples

1.1 Examples of simulation

Several examples of projects are stored in the github Examples directory of the φ-evo package:

- adaptation (reproducing results of [1])
- somite (reproducing results of [2])
- hox and hox_pareto (reproducing results of [3] and of current paper)
- lac_operon (reproducing results of current paper)
- immune (reproducing results of [4])
- minimal_project defines empty function for testing, and is a good starting point for user-defined functions. Notice the Notebook `ProjectCreator` also allows to define other evolutionary parameters such as initial topologies

These project directories contain all necessary files to perform evolutionary runs similar to the ones presented in the main text and in previous studies. They can either be cloned using `git` or downloaded directly using the φ-evo download tools.

To use the download tools, the φ-evo library must be correctly installed. The following python script will download everything required for the evolution of a given project (here the adaptation one) on a local computer:

```
# python
import phievo
phievo.download_example("adaptation")
phievo.download_tools() # Download run_evolution.py and AnalyzeRun.
    ipynb
```

in a python shell (e.g. Ipython).

An evolution is run with the *run_evolution* script. The project path is specified using the *-m* option. The following command

```
# shell
python3 run_evolution.py -m example_adaptation
```

launches an evolutionary simulation with parameters stored in the directory project `example_adaptation`.

Instead of running an existing project one can also create a new project *de novo* as described in the documentation. To simplify the process of starting a new project, the ProjectCreator notebook can be used:

```
# shell
jupyter notebook ProjectCreator.ipynb
```

A short tutorial video explaining how to create a biased version of the somitogenesis project with the `ProjectCreator` notebook is availabe on Youtube.

Once the simulation is finished, the results can be easily analyzed with the help of a jupyter notebook, e.g.:

```
# shell
jupyter notebook --NotebookApp.iopub_data_rate_limit=10000000000
    AnalyzeRun.ipynb
```

A more comprehensive guide on how to analyze the results is available here.

A short tutorial video explaining how to run the code and the analysis on a simple example is available on Youtube.

Immune "add-on"

We include implementation of *in silico* evolution of an immune recognition system similar to [4] as an independent package, where new interactions are defined as external add-ons. This illustrates how new interactions and new problems can be encapsulated for specific problems. A specific Notebook is also included for visualization. Check README in directory `example_immune` for more details. We show one result of the evolution of such network on Figure S 1

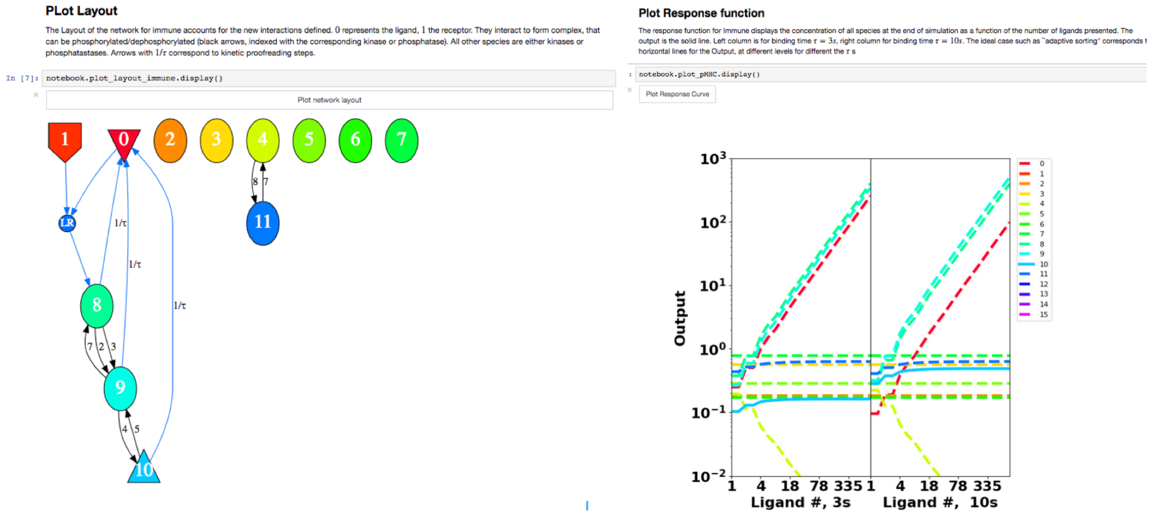

Figure S 1: Notebook output for the evolution of an immune recognition network. (Left) We display the network topology found by φ-evo combining a proofreading cascade (nodes $8 \rightarrow 9 \rightarrow 10$) with the regulation of one kinase. An adaptive sorting motif similar to what was observed in [4] has evolved, where the complex (node 8) formed by the ligand (node 0) receptor (node 1) interaction phosphorylates the kinase (node 4), which is implicated downstream of the proofreading cascade. (Right) The behaviour displayed shows the concentration of all species as a function of the number of ligands presented for two types of ligands (binding times $\tau = 3s$ and $\tau = 10s$): the output concentration (node 10) displays characteristic flat lines at different levels for different binding times, allowing for discrimination.

1.2 Examples of runs

Because some simulations can take time and ressources to run, we also provide the several full evolutionary runs we used to generate the figures of the manuscript:

- seed_adaptation
- seed_adaptation_pruning
- seed_somite
- seed_somite_pruning
- seed_lacOperon

- seed_lacOperon_pruning
- seed_hox_pareto_light

Those runs are stored in "Seed" directories (indicating the random seed used for this specific run). Downloading existing runs is similar to downloading a project:

```
# python
import phievo
phievo.download_example("seed_adaptation")
phievo.download_tools()
```

The results can also be visualized using the `AnalyzeRun.ipynb` notebook or by following the instructions in the documentation.

Hox pareto

The complete simulation for the Hox Pareto taking a lot of disk space, only a portion of the original results is accessible through φ-evo . You can manually download the complete simulation here.

2 Supporting information : Pruning on somitogenesis and adaptation evolution

We simplify the end results of the simulations with an extra step of evolutionary pruning. Starting from the best network obtained from a simulation (somitogenesis or adaptation) the simulation is restarted allowing only edge and node removal and possibly parameter mutations. The purpose of the procedure is to get rid of the unnecessary complexity of the network obtained from the original simulation.

The pruning did not reduce the structure of final network of the adaptation evolution.

On the somitogenesis example, the pruning removed an inhibition from O_3 on O_1. O_1 needs an activation from S_2 in order to be present. A inhibition of O_3 on S_2 is enough to lead to a reduction of O_1 production. Figure S 2 shows the network before undergoing a pruning evolution.

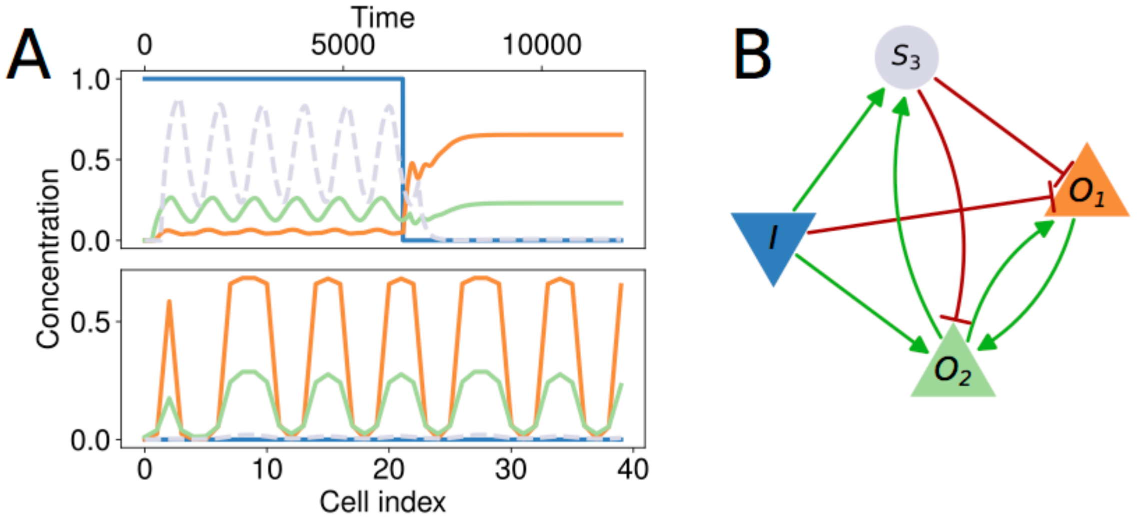

Figure S 2: **A** Time course of the protein concentration in one cell (*top*) and final embryo profile (*bottom*). **B** Network before pruning, the inhibition from O_3 on O_1 is still present.

3 Supporting information – lactose operon

3.1 Choice of the γ parameter

As said in the main text, evolution requires a smooth fitness landscape. It thus depends on the choice of the parameters β and γ, quantifying respectively the benefit and the cost of the protein production.

In a binary framework, there are four possible inputs configuration that we can write in the form of 00, 01, 10 and 11 ($0/1$ corresponding respectively to absence/presence of the Inupt). For each of these four input configurations, taken in this order, we can also express the Output configuration for a given logical gate. For instance, the AND logical gate can be encoded with 0001, meaning that out of the 4 input configurations, the network should respond only to the last one (11).

If we want to evolve such a logical gate, we need to adjust β and γ so that the corresponding fitness is minimal. Fixing $\beta = 1$, and assuming that all input configurations have equal weight (rescaled to 1), we thus expect the following logical gates to have fitness equal to

Logical Gate	truth table	fitness
False	0000	0
True	1111	$3\gamma - 1$
Input1	0101	$\gamma - 1$
Input2	0011	$\gamma - 1$
AND	0001	-1

We see that the best (lowest) possible fitness is obtained for the AND gate as expected by design. But if we want a smooth (downhill) landscape leading to this AND gate, we should ensure that intermediate steps leading to it are favorable, e.g. that gates such as Input1 or Input2 alone should be lower than the non responding gate that is permanently off. This imposes that $\gamma < 1$. The fitness gap between the best and the second best solution being γ, we also choose this parameter as large as possible to have a significant selective pressure. Since it needs to be at the same time significantly lower than 1, $\frac{3}{4}$ represents a good compromise that we take.

3.2 Dynamics of evolution

We present dynamics of the evolution for one successful run on Figure S3. Given the discussion on γ above, we would expect to see two plateaus of fitness for the development of the two branches of the AND regulation (from 0 to $\gamma - 1$ to γ), however we always see only one sudden jump from 0 to the final fitness. When considering the network structure close to the drop in fitness, we actually see that the two parts of the regulatory mechanism appear close to each other in evolutionary time after a long period of unsuccessful trials. Starting at generation 380, it is only a matter of 20 generations to evolve the optimal topology as can be seen on Figure S 3. On other runs, the path can be slightly more involved but usually takes less than 50 generations.

More generally, the evolution of fitness for different simulations typically exhibits a series of sudden burst corresponding to real innovations in terms of topology of the network that are followed by a characteristic relaxation phase where the parameters of the new topology are finely tuned to minimize the fitness.

4 Supporting information – Pareto front for evolution of Hox-like domains

The following calculation generalizes the one originally published in the Supplement of [3] to explain the shape of the Pareto front discovered here by φ-evo .

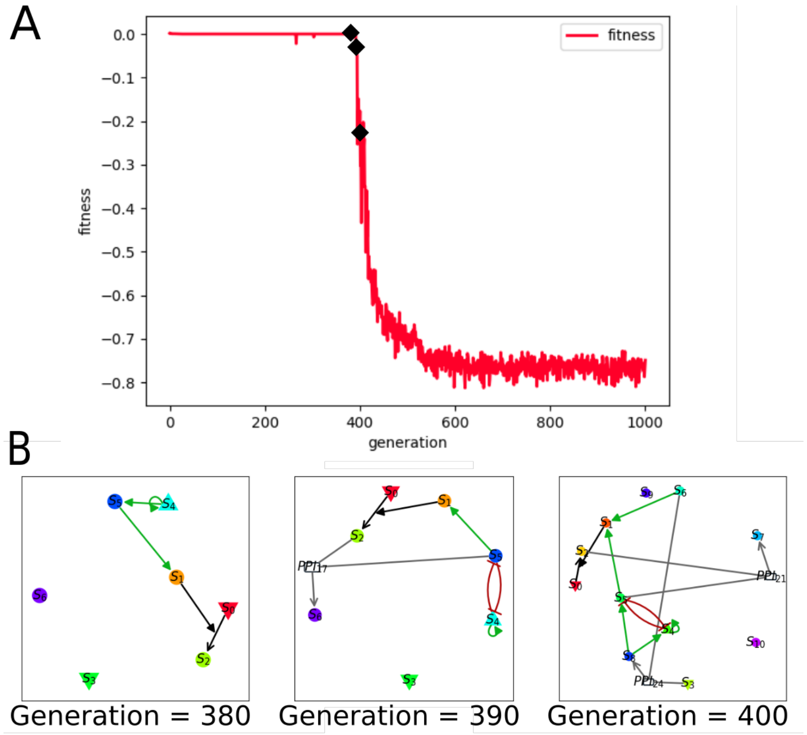

Figure S 3: **A** Example of fitness change as a function of generations for a successful run. There is a clear relaxation of the dynamics where the start of the relaxation is around generation 400 and the characteristic time is around 20 generations.**B** . Three snapshots of the best network in the population at generation 380, 390 and 400 (black diamonds in **A**). It is interesting to see that the two branches of the regulation appear within a small number of generations, thus starting the sudden fitness decrease which is followed by a slow parameter fitting corresponding to the relaxation shape.

The selection should drive the evolution toward networks in which the anterior-posterior axis should present as much diversity as possible in term of gene expression. However one also wants every cell to be specialized and thus to express one or at least only a few genes.

$1^{st} fitness:$ Based on the global entropy

$$-F_1 = \sum_i p_i \log p_i \tag{1}$$

$2^{nd} fitness:$ Based on the local entropy:

$$F_2 = -\sum_x \sum_i p_{i|x} \log p_{i|x} \tag{2}$$

Given the probability definitions:

$$p(i|x) = \frac{1}{L} \frac{c_{i,x}}{\sum_j c_{j,x}} \qquad \text{and} \qquad p(i) = \sum_x p(i|x) \tag{3}$$

With $c_{i,x}$, p_i, $p_{i|x}$ and L being respectively the concentration of protein i in cell x, the global probability of protein i, the local probability of protein i in the cell x and L the number of cells in the embryo. The $1/L$ factor assigns the same weight to every cell.

We consider the simple scenario where only the relative concentrations of the genes already expressed in a region is changed by a mutation. We also assume genes are only expressed in a unique region R of size K cells. Let evaluate a ***global fitness*** as in [3]:

$$F = F_2 - F_1$$

In a the region R where the protein i is expressed, let us rewrite $p_{i|x} = a_i$. For the cells outside of R, $p_{i|x} = 0$. It also leads to $p_i = K a_i$.

$$-F_1^R = \sum_{i \in R} (K a_i \log K a_i) \qquad F_2^R = \sum_{i \in R} (-K a_i \log a_i)$$

The contribution of R to the global fitness is

$$F^R = K \sum_{i \in R} a_i \log K$$

And because the genes are present in only one region, the normalization condition $\sum_{i \in R} a_i = 1$ applies:

$$F^R = K \log K$$

A change in the relative concentrations of the genes present in the region R affects the weights a_i but not the size K. The normalization being still verified, the total fitness F remain unchanged.

As a consequence, a mutation that only changes the relative concentrations of the genes in a given region moves the network on a straight line of slope -1 in the ($-F_1$,F_2) space. This explains why most mutations localize on this Pareto front.

5 Quick introduction on the structure of the networks

Lastly in the following we very briefly review the main types defined in Python to encode the networks. More details can be found in the documentation of φ-evo .

5.1 Species

: `classes_eds2.py`

Parameters: List of species types `types`

List of possible tags. Species may have the following tags (the term in parenthesis indicate the existence of a second parameter attached to the tag and its type):

Species A generic tag, mainly used for internal operation

Degradable (*float* degradation rate), indicate a passive degradation of the species with a rate given as parameter. Note also that only *Degradable* species may be subjected to an active degradation (see 5.3.4) by an enzyme.

TF (*bool* activity), indicate that the species is a possible transcription factor, the activity indicate if it is an activator (1) or a repressor (0).

Kinase mark the species as a kinase, a species susceptible of phosphorylating others.

Phosphatase mark the species as a phosphatase, a species susceptible of dephosphorylating others.

Output (*int* nput), the species is a possible output, the index indicate the order in which the different outputs are chosen, if only one is needed to compute the fitness, the output tagged as 0 will be used.

Input (*int* nput), the species is an input, the index indicate the order in which the different inputs are used by the fitness.

Complexable The species is susceptible to bind to others to form Complex.

Complex The species is the product of a PPI.

Phospho (*int* nphospho), indicates a phosphorylated species the parameters denotes the number of phosphate groups attached.

Phosphorylable Only species with phosphorylated tags can be phosphorylated.

Diffusible (*float* diffusion), the species diffuse between different cells, the diffusion rate is given as parameter.

Species is the most basic type, encoding chemical species in the cell (most often proteins).

5.2 TModule

: `classes_eds2.py`

Parameters: Production rate r `rate`, basal activity b `basal`

As shown in figure 4, TModules are the main elements in the architecture of the genes. They indicate the production rate which can be modulate by activator and the basal production that cannot. They are 'Species' like in that sense that they can only be linked to interactions (usually CorePromoter downstream and TFHill upstream).

A bare TModule (wihtout activation nor repression) produce its species at a rate given by:

$$\frac{dS}{dt} = (\max(r_S, b_S))_{(t-d_S)}\,, \tag{4}$$

where the lower script $(t - d_S)$ indicate that the whole expression is based on the delayed value where the delay d is a parameter of the core promoter (see below).

5.3 Interactions

Interactions are described in separate python module, however they are added manually to the core of the programm in the `Networks/interaction.py`.

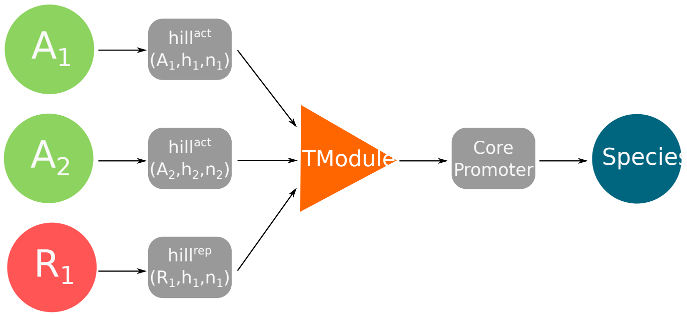

Figure S 4: Inside representation of the gene regulation mechanism by our algorithm, the corresponding equations are given by 5. Note that repressor are multiplicative but only the highest activator is taken into account.

5.3.1 Core Promoter

: `CorePromoter.py`

Parameters: delay d `delay`

Core Promoter are the main element responsible for the addition of a new species in the network. However, as species also need TModule and other technical points, they are only added through the `new_gene` function which create directly the species, the CorePromoter and the TModule (see figure 4). The only parameter of Core Promoter are an integer called delay which determine the time (in number of integration steps) that the new species take to be produced. Thus it is only after a certain time that an activated protein will appear in the system.

5.3.2 Transcriptional Regulation

: `TFHill.py`

Parameters: Activation/repression flag `activity` – Concentration threshold h `threshold` – Hill coefficient n `hill`

Regulation (in the form of activation or repression) is another important function of living system to process information and adapt to their environment. In our algorithm, species which are tag as transcription factor (`TF`) are susceptible to control the production of other species by interacting with their TModule as depicted in figure 4. We use Hill's equation to model these regulations.

When regulated by other species, the production rate of a species S is given by the

following expression:

$$\frac{dS}{dt} = \left(\max\left\{ r_S \times \max\left\{ \frac{A_1^{n_{A1}}}{A_1^{n_{A1}} + h_{A1}^{n_{A1}}}, \frac{A_2^{n_{A2}}}{A_2^{n_{A2}} + h_{A2}^{n_{A2}}}, \dots \right\}, b_S \right\} \times \frac{h_{R1}^{n_{R1}}}{R_1^{n_{R1}} + h_{R1}^{n_{R1}}} \times \dots \right)_{(t-d_S)}, \quad (5)$$

where r_S and b_S are the rate and basal parameter from the associated TModule (see 5.2), A_i denotes the activators, R_i the repressors and the lower script $(t - d_S)$ indicate that the whole expression is based on the delayed value where the delay d is a parameter of the core promoter.

So in plain text, the strongest of the activator multiply the production rate and gives the un-repressed production if it is higher than the basal rate, otherwise, this rate is maintained to the basal level. Then each repressor add a (lower than 1) multiplicative term given by its hill function. Note that when several activators are simultanously present, only the strongest of them is taken into account.

5.3.3 Protein-Protein Interaction

: `PPI.py`

Parameters: Association rate k_{on} (`association`) – Dissociation rate k_{off} (`dissociation`)

Binding among protein are certainly among the most common interactions present in cells. Our protein-protein interaction (usually abbreviated PPI) take two existing species and create a new one corresponding to their complex. (The complex species is created when the PPI is added to the network and automatically deleted when the interaction is removed.).

$$r_a = k_{\text{on}} S_1 S_2 \quad (6)$$

$$r_d = k_{\text{off}} C \quad (7)$$

Given the reactant S_1 and S_2 the complex C is produced at a rate r_a and dissociates back to its reactants at the rate r_d. Note that the complex can then have reaction, degradation, etc. of its own independently of its reactant species.

5.3.4 Degradation

: `degradation.py`

Parameters: Degradation rate r (`rate`)

Each species is suceptible to degrade naturally with a fixed degradation rate δ which is under evolutionary control. However, species can also act as enzyme and promote

actively the degradation of other ones. This interaction called degradation has for single parameter the degradation rate r and linked to an enzyme E and a degraded species S. The degradation rate of S is therefore given by $(\delta + rE) \cdot S$.

5.3.5 Phosphorylation

: `Phosphorylation.py`

Parameters: phosphorylation rate k_p (`rate`) – threshold h (`threshold`) – hill coefficient n (`hill`) – dephosphorylation rate k_d (`dephosphorylation`)

A given kinase K may phosphorylate different species and thus be involved in several phophorylations. The rate at which the species S is phosphorylated into species S^p, is given by:

$$k_p \frac{K\left(\frac{S}{h}\right)^n}{1 + \left(\frac{S}{h}\right)^n + \ldots}, \tag{8}$$

where the dots stands for the other phosphorylations in which the kinase may be involved. The dephosphorylation is supposed to be constant at rate $k_d S^p$. Note that there is no phosphatase in this model.

References

[1] François P, Siggia ED. A case study of evolutionary computation of biochemical adaptation. Physical Biology. 2008;5(2):26009.

[2] François P, Hakim V, Siggia ED. Deriving structure from evolution: metazoan segmentation. Molecular Systems Biology. 2007 Dec;3:9.

[3] François P, Siggia ED. Predicting embryonic patterning using mutual entropy fitness and in silico evolution. Development (Cambridge, England). 2010;137(14):2385–2395.

[4] Lalanne JB, François P. Principles of adaptive sorting revealed by in silico evolution. Physical Review Letters. 2013 May;110(21):218102.
